# Enzymatic Modification of Native Chitin and Conversion to Specialty Chemical Products

**DOI:** 10.3390/md18020093

**Published:** 2020-01-30

**Authors:** Nathanael D. Arnold, Wolfram M. Brück, Daniel Garbe, Thomas B. Brück

**Affiliations:** 1 Werner Siemens Chair of Synthetic Biotechnology, Dept. of Chemistry, Technical University of Munich (TUM), 85748 Garching, Germany; nathanael.arnold@tum.de (N.D.A.); daniel.garbe@tum.de (D.G.); 2 Institute for Life Technologies, University of Applied Sciences Western Switzerland Valais-Wallis, 1950 Sion 2, Switzerland; wolfram.bruck@hevs.ch

**Keywords:** chitin, chitosan, chitooligosaccharides, enzymatic modification, biotechnology, chitinase, chitosanase, lytic polysaccharide monooxygenase, chitin deacetylase

## Abstract

Chitin is one of the most abundant biomolecules on earth, occurring in crustacean shells and cell walls of fungi. While the polysaccharide is threatening to pollute coastal ecosystems in the form of accumulating shell-waste, it has the potential to be converted into highly profitable derivatives with applications in medicine, biotechnology, and wastewater treatment, among others. Traditionally this is still mostly done by the employment of aggressive chemicals, yielding low quality while producing toxic by-products. In the last decades, the enzymatic conversion of chitin has been on the rise, albeit still not on the same level of cost-effectiveness compared to the traditional methods due to its multi-step character. Another severe drawback of the biotechnological approach is the highly ordered structure of chitin, which renders it nigh impossible for most glycosidic hydrolases to act upon. So far, only the Auxiliary Activity 10 family (AA10), including lytic polysaccharide monooxygenases (LPMOs), is known to hydrolyse native recalcitrant chitin, which spares the expensive first step of chemical or mechanical pre-treatment to enlarge the substrate surface. The main advantages of enzymatic conversion of chitin over conventional chemical methods are the biocompability and, more strikingly, the higher product specificity, product quality, and yield of the process. Products with a higher M_w_ due to no unspecific depolymerisation besides an exactly defined degree and pattern of acetylation can be yielded. This provides a new toolset of thousands of new chitin and chitosan derivatives, as the physio-chemical properties can be modified according to the desired application. This review aims to provide an overview of the biotechnological tools currently at hand, as well as challenges and crucial steps to achieve the long-term goal of enzymatic conversion of native chitin into specialty chemical products.

## 1. Introduction

Since the first International Conference on Chitin/Chitosan (ICCC) was held in 1977, the interest in chitin-derived products has increased substantially. It is one of the most abundant biopolymers on our planet, second only to cellulose, occurring in the exoskeletons of crustaceans and insects, the radulae of molluscs, as well as in fungi and algae cell walls, rendering it a readily available resource. The amount of chitin produced every year by living organisms on a global scale is estimated to be in the magnitude of 10^10^–10^11^ tons, of which 10^6^ tons are produced solely by the seafood processing industry in the form of crustacean shell wastes originating from shrimps, prawn, crabs, and lobsters [1]. These secondary products are composites of a stabilizing chitin scaffold interfaced with calcium carbonate, protein, carotenoids, hereby predominantly astaxanthin, and small amounts of lipids. The relation of the respective components is dependent on the source species and seasonal changes. This can be exemplified by sclerotin, the tanned proteinaceous matrix constituting most of the insects’ exoskeletons.

As the global demand for luxury foods increases, large amounts of crustacean shell wastes are generated that are disposed into either the ocean or landfills. Although chitin itself is biodegradable, its natural composites are recalcitrant and barely accessible for naturally occurring enzymes, therefore accumulating over time and threatening coastal ecosystems [2].

## 2. Conventional and Biotechnological Methods of Chitin Extraction and Conversion

### 2.1. Chemical Chitin Extraction

**Figure 1:**
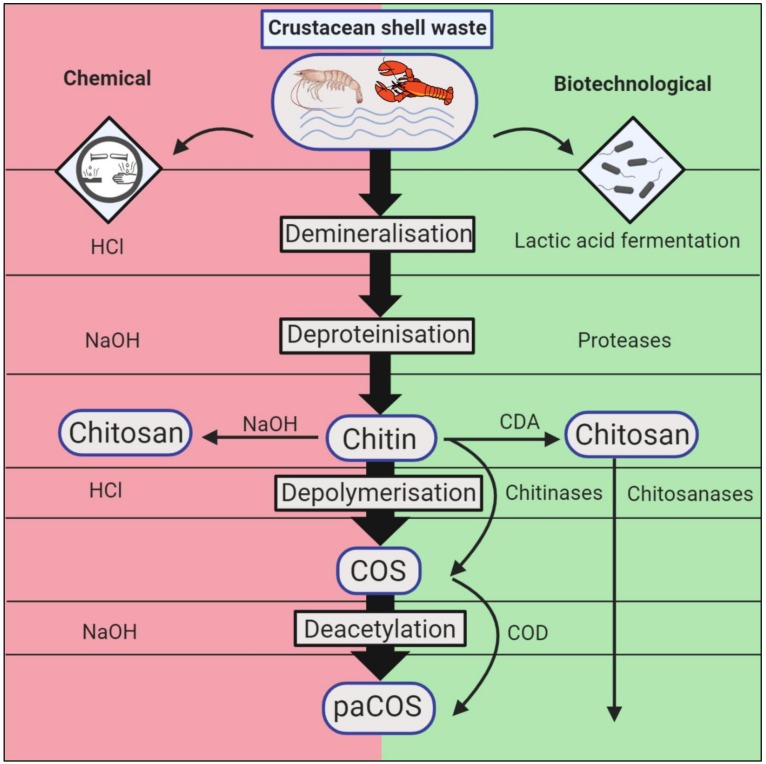
Chemical and biotechnological chitin extraction methods from crustacean shell waste. Since native chitin from marine sources is a composite of calcium carbonate, protein, carotenoids, and small amounts of lipids, it has to be processed for industrial applications. After mincing and cleaning of the crustacean shell waste, it has to be freed of minerals and proteins. This can be achieved chemically (red, left side) by the employment of HCl and NaOH, respectively, while biotechnological approaches (green, right side) utilise lactic acid bacteria fermentation for mineral removal and protease cocktails for the excision of residual protein. To obtain the industrially relevant end products of chitosan and its partially acetylated chitooligosaccharides (paCOS), chitin is depolymerised by acid-hydrolysis or chitinases in a first step. Afterwards, the obtained chitooligosaccharides (COS) are N-deacetylated with 50% NaOH at room temperature or 120 °C, conventionally, or through chitin oligosaccharide deacetylases (COD), enzymatically. If high M_w_ chitosan is desired, the isolated chitin can be converted directly through incubation with hot alkali or chitin deacetylases. The figure was created with BioRender.

The conventional extraction of chitin is performed chemically by demineralising the ground crustacean shells by means of strong acids, favourably HCl, and subsequently removing residual protein through incubation with a strong base, whereby NaOH is the alkali of choice [3,4]. If so desired, the chitin flakes can be bleached by H_2_O_2_, KMnO_4_, or equally strong oxidizing agents in an additional step to remove remaining dyes. The whole process generates hazardous by-products while rendering valuable minerals and amino acids hardly recyclable, yielding chitin with a low M_w_ (molecular weight), since the aggressive chemicals attack the crystalline structure and therefore randomly degrade the co-polymer chains [5] (Figure 1, left side).

### 2.2. Chemical Processing of Chitin into Chitosan

After obtaining mineral- and protein-free chitin from crustacean wastes, with the help of strong mineral acids and NaOH, respectively, the chitin is converted into chitosan by means of 25–50% NaOH-assisted de-N-acetylation. This can be done at room temperature (homogeneous deacetylation) or at elevated temperatures (heterogeneous deacetylation) depending on the desired product, with the latter being the preferred method for industrial needs [1,6]. Afterwards the product is washed to remove the alkali and dried to obtain chitosan flakes. The whole process harnesses randomly deacetylated chitosan chains with varying M_w_ and an unspecific P_A_ (pattern of acetylation), which have to be fractionated according to their physiochemical properties with elaborate, time-consuming methods [7]. Complementing the thermochemical approach, microwave irradiation was successfully used to increase the speed, yield, and degree of deacetylation (D_D_) of chitosan obtained from shrimp shells [8]. El Knidri et al. took this method a step further, utilising microwave irradiation exclusively to produce chitosan, which accelerated the whole process 16-fold [9]. The obtained chitosan had a higher M_w_ with an equal D_D_ of about 80% as that of thermochemically yielded chitosan.

### 2.3. Biotechnological Chitin Extraction

Biotechnological methods to obtain chitin from crustacean shell wastes comprise demineralisation through lactic acid bacteria fermentation and deproteinisation by means of proteolytic bacteria or protease cocktails [10,11,12,13]. Alternatively, protease activity may simply be induced passively by pH reduction in lactic acid fermentation due to conversion of glucose, leading to accumulation of calcium lactate and removal of residual protein [14]. As a consequence, chitin with demineralisation degrees up to 99% and deproteinsation degrees around 95% can be achieved [15], retracting the protein and minerals in a liquid phase without environmentally harming by-products. Both successive two-step fermentations and one-batch co-fermentations have been tried hereby, while the latter approach would reduce costs in an industrial scale significantly [16,17,18]. While the succession of the demineralisation and deproteinisation steps does not negatively affect the quality or yield of chemically processed crustacean shells, it is of importance to perform the demineralisation prior to protein removal, since the minerals can inhibit protease activity [4,19]. The majority of research projects have focused on aerobic fermentation as instrument of choice, requiring aeration and careful monitoring of batch O_2_ contents, which represents an additional cost factor. Bajaj and colleagues demonstrated the efficiency of anaerobic fermentation of shrimp shell waste with simple tools, e.g., minced meat and bio-yoghurt as sources for proteolytic- and lactic-acid bacteria, respectively (Figure 1, right side).

In their outstanding study they were not only able to reuse their fermentation liquor for another large-scale fermentation (300 L), attaining chitin with comparable degrees of deproteinisation, but also produced chitosan with a higher viscosity than commercially available products [20].

Despite the grievous disadvantages of chemical chitin extraction, it still remains the standard procedure in the industry to date, probably due to the faster extraction times.

### 2.4. Biotechnological Chitin Conversion

Biotechnological chitin processing be achieved by applying several enzymes, either to cleave chitin into smaller fragments, the chitooligosaccharides (COS), through chitinases (EC 3.2.1.14) and β-N-acetylhexosaminidases (EC 3.2.1.52), or by conversion into chitosan via chitin-deacetylases, catalysing the hydrolysis of the acetamido group (EC 3.5.1.41). As auxiliary enzymes, lytic chitin monooxygenases (LPMOs, EC 1.14.99.53) may be deployed, as they are able to hydrolyse native crystalline chitin by oxidation. This increases the accessibility for regular chitinases. Chitosan can also be cleaved enzymatically by chitosanases (EC 3.2.1.132) to produce prevalently deacetylated COS.

## 3. Characterisation of Chitin, Chitosan, and Chitooligosaccharides

### 3.1. Chitin

**Figure 2:**
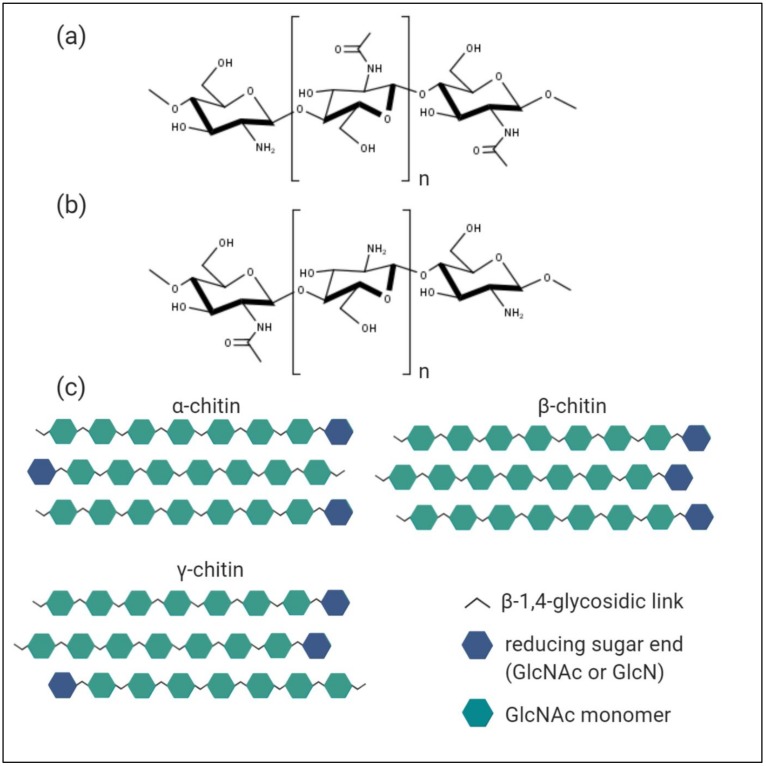
Chemical structures of chitin and chitosan. (**a**) Chitin is a homo-polymer assembled of N-acetyl-D-glucosamine monomers (GlcNAc), covalently bound through β-(1-4)-glycosidic linkages. In nature, it occurs as a co-polymer comprised of both GlcNAc and non-acetylated D-glucosamine (GlcN) subunits, with a molar GlcNAc fraction > 50%. (**b**) Chitosan is the deacetylated derivative of chitin, defined by a ratio of GlcN to GlcNAc monomers of > 1,0. It exhibits solubility in aqueous acetic solvents. (**c**) Chitin has three different allomorphs, which differ in the orientation of the respective polymer chains within the micro-fibril macro structure. The most abundant and resilient α-chitin is formed by antiparallel aligned polysaccharide chains. In β-chitin, the sugar chains are ordered in a parallel manner, therefore exhibiting weaker intramolecular interactions. The γ-allomorph of chitin is characterized by a mixture of both antiparallel and parallel aligned chains, which leads to a polymer with fractions of higher and lower levels of crystallinity. The figure was created with BioRender.

The homo-polymer chitin is assembled by units of N-acetyl-D-glucosamine (GlcNAc) covalently bound through β-(1-4)-glycosidic links, possessing a high M_w_ (Figure 2a). The polysaccharide has a lot in common with cellulose structurally, the difference is due to acetamido groups substituting for the hydroxyl groups at position 2 of the respective 2-deoxy-D-glucopyranose-subunits. In nature, chitin exists as a co-polymer comprised of randomly distributed GlcNAc and D-glucosamine (GlcN) subunits, in which the acetylated D-glucosamines prevail. Chitin is not soluble in aqueous solutions due to its high hydrophobicity and resembles a rigid, inflexible crystalline structure, since the acetamido groups form strong hydrogen bonds between adjacent polymer chains, which again are ordered in micro-fibrils. The polysaccharide is primarily found as part of composite materials, where it acts as a stabilizing scaffold interfaced with minerals like calcium carbonate, protein, or both.

The strength of the inter- and intramolecular interactions within the chitin micro-fibrils is dependent on the orientation of the polymer chains, which is manifested in the three naturally occurring allomorphs of α-, β-, and γ-chitin [1] (Figure 2c). The most abundant and resilient α-form of chitin is characterized by the antiparallel alignment of the polymer chains; it occurs in all exoskeletons of arthropoda. In contrast, within β-chitin the polymer chains are lining up in a parallel manner, exhibiting weaker intramolecular interactions and therefore projecting more accessibility for enzymes. The most prominent source would be squid pens, although the β-allomorph is also present in other molluscs like krill. Structurally γ-chitin differs through a mixture of both parallel and antiparallel alignment of interspersed polymer chains, leading to fractions with higher or lower crystallinity levels, respectively, and it naturally occurs in beetle larvae and cephalopods.

Due to these inherent chemical and physical characteristics of chitin its commercial applications in the native form are currently rather limited. Commonly native chitin is utilised as fertilizer in agriculture, as it contains nitrogen, or as an inducer of plant defence mechanisms, enhancing the resistance against fungi by the promotion of chitinases [21,22,23]. Furthermore, chitin is employed as a food additive [24] as well as a packaging material for affinity chromatography columns to purify proteins with a carbohydrate-binding domain, or as sorbent to pre-concentrate phenol and chlorophenols by means of solid phase extraction, as validated by HPLC [25,26].

### 3.2. Chitosan

The abundant polysaccharide chitin finds an increased number of applications once it is converted into its deacetylated form chitosan, which is defined by a ratio of GlcN to GlcNAc units > 1.0 in the random copolymer [4] (Figure 2b). This transfers into the molar fraction of N-acetylated glucosamine monomers, which represents the degree of acetylation (D_A_), also commonly expressed in percentage (%D_A_).

In research the term chitosan is defined more loosely and comprises hetero-polymer chains with a minimal degree of deacetylation (D_D_) of about 10%, while only fully acetylated chains, exclusively assembled from GlcNAc, are being referred to as chitin. Generally, chitosan is less abundant in nature compared to chitin, occurring only in cell walls of *Zygomycetes* fungi species [27].

The conventional conversion of chitin into its functionalised form chitosan is realised by means of 50% w/w NaOH at high temperatures (80–120 °C) with a 1:10 solid to liquid ratio to hydrolyse the acetamido groups [6]. This yields a chitosan product with a low degree of polymerisation (DP), an undefined D_A,_ in addition to an unspecific pattern of acetylation (P_A_). All of the aforementioned factors combined are critical for the physio-chemical properties and bioactivity of chitosan.

Chitosan is soluble in weakly acetic solutions and demonstrates antibacterial, antifungal, antioxidant, anti-inflammatory, and antitumoral activities, besides being physiologically inert and biodegradable [28,29,30]. Moreover, it exhibits a cationic nature in acetic solution, which is unique among polysaccharides, therefore having the ability to bind to negatively charged surfaces [31,32]. This feature is assumed to be responsible for its antibacterial appeal, either binding to the surface of bacteria, therefore blocking their metabolism or alternatively through attachment of smaller chitosan fragments to the negatively charged DNA, effecting the inhibition of RNA translation [30,33].

### 3.3. Chitooligosaccharides

Correspondingly to chitin, the high M_w_ and viscosity of chitosan, in addition to its low solubility in water, hinder easy processing for industrial applications. Therefore, chitin or chitosan are generally depolymerised either chemically, mechanically, or by means of enzyme degradation to obtain smaller fragments, so-called chitooligosaccharides (COS) or partially acetylated chitooligosaccharides (paCOS), which are soluble in water while exhibiting the same positive traits as their highly polymerised source materials [34]. These COS possess a DP between 2–20 and varying D_A_, P_A_, and F_A_ (fraction of acetylation), which determine their respective biological activity [35,36]. The degree of solubility increases with the D_D_ and is higher for chitooligosaccharides (COS) with a relatively lower DP and a M_w_ up to 3.9 kDa [37]. Chitooligomers with a high DP above 6 and low M_w_ are thought to be more biologically active than COS with a low DP and high M_w_. As are all sugars, chitooligosaccharides are sensitive to autooxidation and thus should be stored at preferably -20 °C under dry and inert conditions. Their shelf life can be increased significantly when antioxidants are added prior to storage [38].

Hence it is a highly attractive biomolecule for various industry segments, from cosmetics and biomedicine to wastewater treatment, textile and paper production, biotechnology as well as the food and agricultural industries. We refer to several excellent reviews that address possible applications of chitin and its derivatives extensively [22,39,40,41,42,43].

To support the increased interest in chitosan derivatives with numbers: its global market is expected to grow at a CAGR (Compound Annual Growth Rate) of 6.3% over the next five years, while exceeding 118,000 tons [44,45]. Also taking into account that the traditional ways of extracting and converting native chitin into its functionalized derivatives are done chemically under toxic waste production, it becomes clear that there exists an urgent necessity to find a suitable biotechnological approach to process crustacean shells by means of enzymes. Not only does this ameliorate the disposal issues for seafood processing companies, but, in doing so, the unused potential of basically free industrial feedstock can be exploited to create specialty chemical products in a more sustainable way with a higher degree of quality [46].

## 4. Characterization of Enzymes Acting on Chitin and its Derivatives

### 4.1. Chitinases

Chitinases (EC 3.2.1.14) belong to the group of glycosyl hydrolases (GHs) and are spread across the GH families 18 and 19, while the family of GH20 contains additional chitinolytic enzymes. These include β-N-acetylhexosaminidases (EC 3.2.1.52), usually referred to as chitobiases, which catalyse the breakdown of dimeric GlcNAc-units (chitobiose) into monomers from terminal-reducing or non-reducing ends of chitin or chitindextrin. Although they all hydrolyse glycosidic β-(1,4)-links of acetylated D-glucosamine units, substantial differences in the mode of action, the amino acid sequence and catalytic sites do exist. While the catalytic regions of GH18 and GH20 glycosidases are characterized through a triosephosphate isomerase (TIM) barrel (β/α)_8_ domain, chitinases from GH19 have a α-helix rich lysozyme-like domain. The family of GH18 is divided into the subfamilies A and B, of which the first additionally possesses a chitinase insertion domain (α + β), or CID, inserted between the seventh α and β-strand of the TIM barrel. The CID consists of five or six anti-parallel β-strands and one α-helix and is putatively responsible for the tunnel-like catalytic cleft and the processive mode of action of the subfamily A chitinases of GH18.

Both enzyme families share a conserved DxDxE motif on the fourth β-strand and catalyse the hydrolytic reaction by a substrate-assisted mechanism [47].

#### 4.1.1. Processive and Non-Processive Chitinases

A chitinase is described as acting processively if it does not release its substrate after hydrolysing a glycosidic link but rather catalyses several cycles in succession while remaining attached to the solid substrate. This is possible by the threading of single chitin chains through the tunnel-like catalytic cleft, while cleaving off disaccharides simultaneously [48]. Non-processive chitinases, on the other hand, detach after every single hydrolysis round, subsequently reattaching to another GlcNAc–GlcNAc link located elsewhere, in a random fashion [49]. Processivity is important to hydrolyse crystalline chitin, maneuvering the enzyme close to a free polysaccharide chain end at the cost of reaction speed [49,50]. Moreover, through the permanent attachment of enzyme and substrate, reattachment of hydrolysed chitobioses is prevented, therefore increasing efficiency.

#### 4.1.2. Endo- and Exo-Chitinases

Chitinases can be specified by their endo- or exo-modus operandi on chitin. The determining factor hereby is the product length formed through chitin hydrolysis. Endo-chitinases cleave glycosidic bonds randomly along the polymer chain in a non-processive manner, producing predominantly chitiobioses, but also other low-M_w_ multimers like chitotrioses (GlcNAc)_2_ or chitotetraoses (GlcNAc)_4_. Exo-chitinases catalyse the degradation of crystalline chitin from the reducing or non-reducing end processively, releasing N, N′-diacetylchitobioses successively, while remaining attached to the substrate. Interestingly, it is always the second and not the first glycosidic β-(1,4)-link that is attacked by exo-chitinases, thereby releasing chitobiose blocks. Another type of exo-acting enzyme would be the β-N-acetylhexosaminidases, which hydrolyse soluble oligomeric degradation products of endo-chitinases into monomeric GlcNAc, targeting the non-reducing end [51]. As their name implies, they catalyse the hydrolysis of hexoses in both gluco- and galacto-configurations [52]. Data suggest that the exo-processing manner of some chitinases is connected to the inaccessibility of crystalline chitin micro-fibrils rather than the enzyme architecture per se, potentially making obsolete the definition by means of product length. Sikorski et al. showed with the example of *Serratia marcescens* chitinases A and B (*sm*ChiA and *sm*ChiB)—two enzymes thought to be exo-chitinases—how, through repositioning of the ‘roof’ in the tunnel-like substrate binding groove, the substrate chitosan could be processed in endo-fashion by the same enzymes [53].

#### 4.1.3. Chitinases with Transglycosylation Capabilities

Intriguingly, several chitinases of family 18 were discovered in the recent past that are capable of not only breaking glycosidic bonds in chitin but likewise of creating new links between small polysaccharide fragments, thus producing longer COS with a DP of up to 13. Enzymatically producing COS with DPs > 4 resembles one of the major limitations currently, since the main products of chitinases are prevalently either dimeric or monomeric, given that the incubation time is long enough. However, these higher polymeric COS hold more value for biomedical and other advanced applications (such as antioxidants with antimicrobial activity in the food and cosmetics industry [30,41,54], biofungicides [55,56], wound healing-agents [57,58,59], in gene therapy [60,61,62], as immuno-stimulants in tumour therapy [63,64,65] etc.), while shorter COS can be harnessed for low value products like fish feed or fertilizer exclusively. The transglycosylation (TG) reaction takes place when a retaining enzyme induces the transfer of a glycosidic residue instead of a nucleophilic water molecule from a donor to an acceptor sugar molecule [51,66]. Madhuprakash et al. solved the crystal structure for *Sp*ChiD of *Serratia proteamaculans*, a chitinase exhibiting both hydrolysis and TG activity. They unravelled how a 13-residue protruding loop in the deep active binding cleft blocked a large number of subsites and suggested this structural characteristic to be responsible for the TG aptitude [67]. Furthermore, the majority of dual action mode chitinases seem to exhibit aromatic amino residues in their catalytic site, suggesting a processive mechanism [67,68]. The exact mechanism of TG events is not fully understood, however for the catalysis of disaccharides it could be shown that the TG is autocondensation-driven and has to happen faster than the glycosidic hydrolysis [69]. Consistent evidence from several studies illustrates how the transfer of COS is dependent on the amount and length of substrate and the proportion and nature of the enzyme. Hereby an excess amount of substrate appears to be beneficial for a TG activity, while a surplus of the glycosidase seems to push the reaction equilibrium towards a predominant hydrolysis agitation [66,68,70].

### 4.2. Chitosanases

Chitosanases (EC 3.2.1.132) are distributed among the families GH5, GH7, GH8, GH46, GH75, and GH80, illustrating their extensive sequence diversities. Another relevant family when talking about chitosan active enzymes would be GH2 with its exo-1,4-β-D-glucosaminidases, which hydrolyse D-glucosamine residues of chitosan and chitosanoligosaccharides from the non-reducing termini successively [71,72]. The endochitosanase family members of GH46 have been studied the most thoroughly, with four crystal structures available and various expression and site-directed mutagenesis studies to encode their mechanism [73,74,75]. To generalize, GH46 chitosanases are mainly built out of α-helices with two globular domains, a minor and major lobe, divided by a deep substrate binding cleft [76,77]. Hereby their core structure resembles that of the well-studied *Escherichia coli* bacteriophage T4 lysozyme (GH24) and to a lesser extent lysozymes belonging to the families GH22,23 and barley chitinases of GH19, in spite of low sequence homology. This catalytic and substrate binding site is constituted of two α-helices and a three-stranded β-sheet and has presumably developed from divergent evolution [78]. Hence, these five aforementioned families are also referred to as the lysozyme superfamily.

While the families of GH5, 7, and 8 comprise enzymes with several other activities like cellulases, xylanases, and glucanases, the families of GH46, 75, and 80 consist of chitosanases exclusively, which are thought to act by an inverting mechanism [79,80]. The inverting reaction mechanism describes a one-step, single-displacement hydrolysis of a glycosidic bond, which results in the net inversion of the anomeric configuration, assisted by a general base and general acid in the form of amino acid side chains [81]. The retaining reaction mechanism on the other hand comprises a two-step, double-displacement hydrolysis of a glycosidic bond, which results in the net retention of the anomeric configuration. Analogously, it is also assisted by two amino acid side chains, which act as both acid/base respectively and nucleophiles to catalyse the process [82]. Both pathways involve oxocarbenium ion-like transition states, whilst the retaining reaction mechanism also includes an additional covalent glycosyl–enzyme intermediate.

Chitosanases are further classified into the subclasses I, III, and IV based on their substrate specificity [83]. All chitosanases share the ability to hydrolyse glycosidic β-(1,4)-linked GlcN-GlcN, although the majority are able to recognize either GlcN-GlcNAc (subclass III) or GlcNAc-GlcN (subclass I) bonds as well, but not both [84,85]. Subclass II chitosanases are restricted to solely recognising and cleaving GlcN–GlcN links. Only chitosanases of the subclass IV are by definition able to cleave both GlcNAc–GlcN and GlcN–GlcNAc, as well as GlcN–GlcN [86]. Moreover, chitosanases have in common an inability to recognize GlcNAc–GlcNAc links in partially acetylated chitosan, although evidence against the strict distinction between chitinases and chitosanases has been unravelled. Hegsett et al. found a glycoside hydrolysing enzyme in *Streptomyces coelicolor*, which they referred to as A3(2), that was able to cleave GlcN–GlcN, GlcNAc–GlcN links and—untypically for chitosanases—GlcNAc–GlcNac, albeit at a much slower rate [87]. Since GlcNAc–GlcNAc and GlcN–GlcNAc dimer products, both of which had to be the result of two independent cleavage events of the hexamer substrate, were not detected after prolonged incubation time, two possible explanations persist. Either A3(2) has an absolute specificity for GlcN subunits at the subsite -2 or it cannot cleave GlcN–GlcNAC links at all.

### 4.3. Chitin Deacetylases

Chitin deacetylases (CDAs, EC 3.5.1.41) and chitooligosaccharides deacetylases (CODs, EC 3.5.1.105) are able to de-N-acetylate chitin and COS respectively, hydrolysing their acetamido groups. They belong to the carbohydrate esterase family 4 (CE4), which also comprises acetyl xylan esterase, peptidoglycan GlcNAc deacetylase, and peptidoglycan N-acetylmuramic acid deacetylase. Structurally, these enzymes share a NodB homology domain, named after the NodB oligosaccharide esterase. The latter is one of the first enzymes to be discovered in the CE4 family, being involved in the nod factor biosynthesis of synergistic *Rhizobium* bacteria. The de-N-acetylation reaction mechanism is proposed to be a nucleophile attack by the conserved Asp–His–His catalytic triad of the CDA on the carbonyl carbon. To date, five different catalytic motifs of CDAs are known, each containing conserved histidine and aspartic acid residues [88]. To elucidate in detail, a catalytic water molecule is at first bound through the metal cofactor Zn^2+^ (in rare cases also Cu^2+^). Secondly, a proton from this water molecule is withdrawn by the catalytic base aspartic acid, therefore creating a nucleophile to attack the carbonyl carbon in the substrate. This results in the intermediate tetrahedral oxyanion, whose negative charge is stabilized by the metal cofactor, which is then protonized by the catalytic acid histidine on the nitrogen [89]. Thereby the free amine group is released alongside an acetate molecule on the zinc.

**Figure 3:**
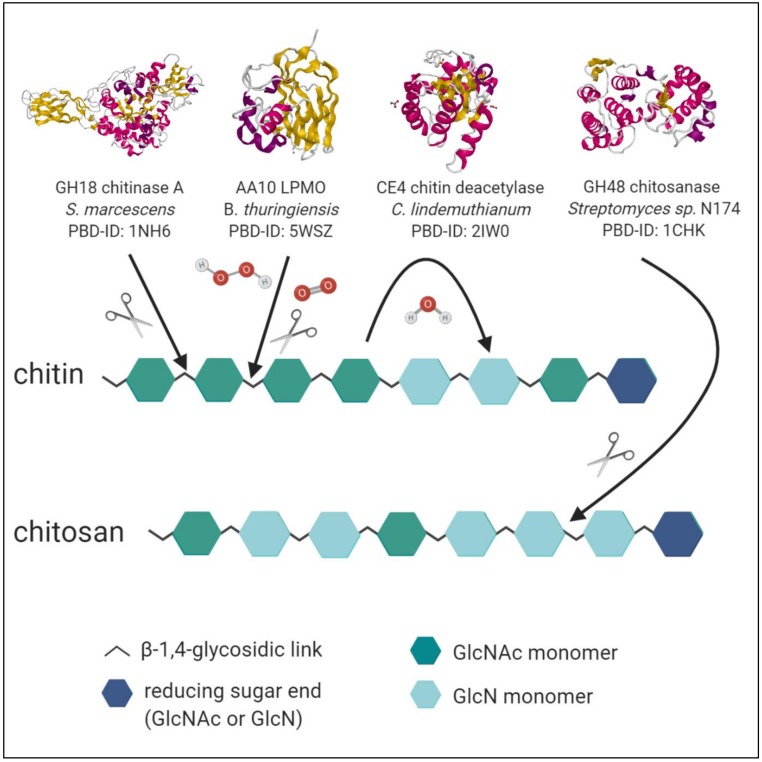
Enzymes with catalytic activity towards chitin and chitosan. The crystal structures of the chitinase A from *Serratia marcescens* [95], the lytic polysaccharide monooxygenase (LPMO) from *Bacillus thuringiensis* [96], the chitin deacetylase (CDA) from *Colletotrichum* *lindemuthianum* [97] and the chitosanase from *Streptomyces* sp. N174 [77], derived from the RCSB protein data bank (PDB), are illustrated as exemplary enzymes of their respective catalytic activity. Red coloured parts are α-helices, while β-sheets are indicated by yellow. Chitinases generally hydrolyse the β-(1,4)-glycosidic links between two GlcNAc monomers. Their modes of action, amino sequence, and catalytic sites can vary substantially. LPMOs are copper-dependent enzymes, which cleave chitin by oxidation of C1 or C4. They can recruit either H_2_O_2_ or O_2_ as co-substrate and need an external reducing agent. The NodB-related CDAs hydrolyse the acetamido groups of GlcNAc monomers with a catalytic water molecule. Each enzyme exhibits specificity for target substrate sequences and creates unique deacetylation patterns. Chitosanases generally hydrolyse the β-(1,4)-glycosidic links between two GlcN monomers with a retaining or inverting mechanism. The specificity towards an additional substrate besides GlcN-GlcN is common. The figure was created with BioRender.

Living organisms take advantage of CDAs both intracellularly, for instance, fungi like *Mucor rouxii* for cell wall morphogenesis or to protect themselves from hostile chitinases [90]; or extracellularly, as observed in maritime bacteria which secrete the enzymes to convert and hydrolyse crustacean shell wastes in succession [91]. Interestingly, the CDAs and CODs are not only highly substrate specific, relying on different recognition patterns, but additionally generate products with specific D_A_ and P_A_ [88]. The resulting D_A_ is hereby dependent on the mechanism of the respective CDA, which can be one of the following three:

1. Enzymes with the multiple attack mechanism bind to their respective recognition site in the polysaccharide chain and process a number of sequential deacetylations, after which they detach and bind to a different region. In the case of high M_w_ substrate so-called block–copolymer structures arise, in which several adjacent, consecutive GlcN subunits alternate with regions of ambiguous D_A_ or GlcNAc, where the CDA is not active. Shorter polymer chains or COS become deacetylated entirely [92].
2. Enzymes following the multiple chain mechanism tightly bind to their recognition site of the substrate, resulting in an enzyme–polymer complex. In contrast to the multiple attack mechanism, the complex already dissociates after a single deacetylation reaction, with the enzyme binding to another recognition site afterwards [93]. For polymeric substrates this results in random distribution of deacetylated subunits and therefore P_A_. For COS no obvious proposition can be made, depending on the specific substrate length and involved enzyme; either a full deacetylation or a specific pattern can be generated. It is of peculiar interest to discover CDAs with new and unique patterns of deacetylation, since the influence of P_A_ on the physio-chemical properties of paCOS remains to be further elucidated in detail. Furthermore, the discovery of more CDAs might also benefit our understanding of their modes of action and in doing so, our ability to tailor chitin- and chitosan oligomers with defined D_A_ and P_A_.
3. Ultimately, single-chain-acting CDAs are processive enzymes that deacetylate a single substrate molecule sequentially by means of several catalytic events. Some bacterial CODs, which are specific for a single position, resulting in mono-deacetylated products (e.g., *Rhizobium* sp. NodB or *Vibrio* sp. CDA and COD) also belong to this group [94]. See Figure 3 for an overview of enzymatic activities of chitinases, LPMO, CDA and chitosanases towards chitin and chitosan polymer chains.

The fact that carbohydrate esterases are highly inactive on crystalline substrates, preferring soluble derivatives like glycol chitin, chitosan, or COS/paCOS, represents a significant drawback to their applicability in the biotechnological conversion of crustacean shell wastes. Interestingly, this can be counteracted by the co-application of LPMOs, which cleave polymer chain ends on the surface of crystalline chitin, as a result enhancing the activity of CDAs [98]. Moreover, several CDAs and CODs are known to possess a carbohydrate binding module (CBM) fused to their catalytic domain, therefore increasing the accessibility of the recalcitrant substrate to the active site [99].

### 4.4. Lytic Polysaccharide Monooxygenases

Lytic polysaccharide monooxygenases (LPMOs, EC 1.14.99.53) are classified into the six auxiliary activity enzyme AA families 9–16 based on sequence, while chitin-processing LPMOs are distributed to the groups 10, 11, and 15. These copper enzymes are characterized by the ability to disrupt the crystalline structure of polymeric polysaccharide substrates by oxidation of the glycosidic link, thus creating new chain ends for common hydrolases to act upon recalcitrant carbohydrates. They are the only enzymes known to date to act on recalcitrant polysaccharide, which confers them a key role in the utilisation of crustacean shell wastes as a chitin source. Furthermore, all LPMOs share a unique histidine–brace motif inside the catalytic region, which is located close to the surface, allowing them to tightly bind a single copper ion by two conserved histidine residues [100,101]. The solvent-exposed flat architecture of the active site is the reason for the ability of some LPMOs to oxidize the crystalline lattice structure of insoluble substrates like chitin or cellulose. The structure of the catalytic region hereby differs amongst different LPMOs, since loops that shape the substrate contact surface are responsible for conferring substrate specificity and regioselectivity. Both single catalytic domain- and multidomain proteins have been discovered, mirroring the high substrate promiscuity of LPMOs. Similar to CDAs, some LPMOs are known to have a C-terminal CBM fused to their catalytic domain with a short linker, further boosting the substrate specificity and binding affinity [102,103]. Since LPMOs do not actively cleave glycosidic linkages inside polysaccharide chains, the nomenclature may be misleading concerning its function, and therefore several researchers proposed the name polysaccharide monooxygenases (PMOs) instead. The cleavage of the glycosidic bond happens rather spontaneously, after the strong C–H bond of either C1 or C4 is deprived of one hydrogen atom and subsequently hydroxylated by the LPMO with O_2_ or H_2_O_2_ as co-substrate. To increase the binding affinity to the polysaccharide and to activate the co-substrate, reduction of the active site Cu(II) to Cu(I) is required [104]. Depending on the co-substrate and the attacked carbon atom, different reaction mechanisms occur. Given that the C1-positioned carbon atom of the glycosidic bond is oxidized, aldonolactone products occur that hydrate to aldonic acids, while C4 oxidation leads to the formation of 4-ketoaldose products that hydrate to *gem*-diol [105,106,107].

#### 4.4.1. LPMO Mechanisms with O_2_ as Co-substrate

When O_2_ is utilized to produce an oxidizing species strong enough to hydroxylate the glycosidic bond, the oxygenase reaction happens regioselectively without inactivation of the enzyme itself [108]. Under absence of substrate and H_2_O_2_, the Cu(I) active state LPMO readily reduces dioxygen, thus generating H_2_O_2_ while transferring the enzyme in its Cu(II) resting state; this is also referred to as futile cycle [109]. The addition of polysaccharide influences the reaction kinetics fundamentally, leading to a coupled hydroxylation reaction of the polysaccharide with O_2_. Hereby two reaction mechanisms are proposed: in the first one, the Cu(I) active state LPMO reacts with O_2_ and an additional electron as well as two additional protons provided by external reductants, ending in the Cu(II) oxidation state after every cycle. Since the hydrogen atom has to be abstracted before the delivery of the electron and two protons for this mechanism to work, Cu(II)-O_2_^•-^ is most likely the oxidizing species [110,111]. In the second mechanism, one external electron donor is necessary again to render the LPMO in its Cu(I) catalytic state initially. Thereafter, two electrons and protons have to be provided, which leads to the heterolytical cleavage of the O–O bond in the dioxygen co-substrate and as a consequence, the formation of a Cu(II)-oxyl oxidizing species (Cu(II)-O^•^). Following the hydroxylation of the polysaccharide, the Cu(I)-state enzyme is regenerated, ready for another monooxygenase reaction cycle [112].

#### 4.4.2. LPMO Mechanisms with H_2_O_2_ as Co-substrate

A study indicated that H_2_O_2,_ and not molecular oxygen, is the preferred co-substrate of LPMOs in a peroxidase reaction with polysaccharides [113], with a 26-fold faster reaction speed and the ability to outcompete O_2_ in the presence of chitin substrate. Contradictive arguments would be the non-regioselective oxidation mode of polysaccharide chains in presence of H_2_O_2_ in addition to the inactivation of the enzyme itself as result of detrimental self-oxidation events [108,114]. Moreover, when peroxide was applied to abduct H_2_O_2_ as co-substrate in an experimental setup of the *Neurospora crassa* secretome and a commercial cellulose gel, no negative effect on the hydrolysation could be observed, thus impeaching its physiological contribution [108]. Interestingly enough, carbohydrates possess the ability to scavenge H_2_O_2_, which could explain the undirected oxidation of glycosidic bonds, while at the same time ameliorating self-inactivation of the LPMO when exposed to higher than micro-molar concentrations of H_2_O_2_, which they can resist. Hereby the correct ratio of H_2_O_2_ to saturating amounts of substrate are of utmost importance to guarantee a sustainable processivity, since the LPMOs are protected of self-oxidation as long as substrate is bound to the active site [114]. 

Analogously to the O_2_-utilizing reaction mechanism, the LPMO catalytic centre has to be pre-reduced into its Cu(I) oxidation state when harnessing H_2_O_2_ as co-substrate. Promising candidates for external electron donors of bacterial PMSOs are oxidoreductases or other redox-active enzymes [115]. Since H_2_O_2_ constitutes a two-electron reduced O_2_ species, no additional external electron source is required after the initial priming reduction to regenerate the Cu(I)-LPMO after each catalytic cycle. The reactivity of H_2_O_2_ might rely on the same Cu-oxyl oxidizing species as that with O_2_, omitting a Cu(II)-OOH intermediate. Computational evidence supports a Fenton chemistry-like production of ^•^OH radicals as means of Cu-oxyl intermediate formation [116], while the heterolytic cleavage of the O–O bond of a Cu(I)-OOH intermediate has also been proposed as alternative pathway [108]. Recently a copper-dependent particulate methane monooxygenase (pMMO), involved in the methane-to-methanol oxidation, was found to have a similar active site architecture and potential mechanism as LPMOs [117], representing another stepping stone in deciphering the highly interesting class of auxiliary enzymes.

## 5. Pretreatment of Native Chitin and COS Synthesis

The central constriction of straightforward COS synthesis from native chitin is its crystalline tertiary structure, resulting from the parallel or anti-parallel packaging of single saccharide chains, which again are ordered as a micro-fibril superstructure. As a consequence, most chitinolytic enzymes can barely access and thus catalyse the recalcitrant substrate, making a pretreatment inevitable. For research purposes, e.g., screening for chitinolytic microorganisms or enzymatic activity assays, it is a common practice to prepare either so-called colloidal chitin (CC) or swollen chitin by means of hydrochloric acid or phosphoric acid treatment, respectively, to increase the substrate surface and solubility. For large-scale industrial purposes on the other hand, this is neither economically nor environmentally feasible. Several endeavours have been undertaken to overcome this hurdle, ranging from physical and mechanical to chemical and lastly, biotechnological pathways.

### 5.1. Physical Pretreatment Methods and COS Synthesis

The most obvious method would be the mechanical grinding of crustacean shell wastes, which as a matter of fact represents the starting point of processing, whether it is chemical or biotechnological. In doing so, reliable removal of minerals and meat residues is ensured. However, experience has shown that this method alone is not sufficient for common chitinases to hydrolyse the rigid substrate flakes. To advance the general idea of a mechano-chemical breakdown, Nakagawa et al. developed a “converge” ball mill, generating fine chitin particles with reduced crystallinity, which they could convert afterwards into GlcNAc or GlcNAc_2_ by means of commercial enzyme mixes with high efficiency [118]. When chitosan is depolymerised in aqueous solution to obtain paCOS, salt formation is inevitable, diminishing the range of applications. This particular drawback can be circumvented by mechano-chemical grinding prior to enzymatic conversion [119].

Other studies examined the effects of gamma irradiation, microwaving, steam explosion, and low ultra-frequency sonification on the structures of chitin and chitosan. These physical approaches predominantly lead to partially depolymerised polysaccharide chains with a hardly controllable range of product M_w_, herein often times short COS with low DP (1-4), while consuming large amounts of energy or right-out damaging the structural integrity of chitin oligomers, rendering them inconvenient for industrial mass production [120,121,122,123,124,125] chemical chitin pretreatment.

Since the chemicals, which are deployed to both extract and successively convert chitin into chitosan (50% NaOH at high temperatures for several hours) and lastly process it into paCOS (35% HCl at 80 °C for short amount of time), are very strong bases and acids, no additional pretreatment is applied on an industrial level. In a chemoenzymatic approach, chitin will be processed with phosphoric acid into swollen chitin or hydrochloric acid into colloidal chitin to increase the accessibility and solubility for a following enzymatic degradation into COS.

Recent studies investigated the chemical pretreatment of chitin by means of so-called ionic liquids (ILs), salts with a melting point below 100 °C and natural deep eutectic solvents (NADES), which consist of quaternary ammonium salts with a hydrogen bond donor-like amines or carboxylic acids. Both of these solvents have a low vapour pressure, rendering them inflammable, and are chemically inert, reusable, and stable. However, IL are more expensive and toxic in comparison to the biologically degradable NADES, which are assembled of primary plant metabolites (amino acids, sugars, and carboxylic acids) occurring in the tissue of living organisms [126,127,128,129]. Interestingly enough, ILs can solubilise polysaccharides into a hydrogel-like amorphous substance without decreasing the DP, therefore increasing the amount of enzyme binding sites presented and overall conversion rate up to 90-fold as shown for cellulose [130]. Zhu et al could extract chitin directly from lobster shells catalysed by NADES consisting of mixtures of choline chloride (vitamin B_4_) and mild carboxylic acids like malonic and lactic acid in different ratios, obtaining a 20% yield [131,132]. Two subgroups with different degrees of crystallinity of the resulting chitin could be distinguished, both of which projected a porous structure. These results are promising, albeit no further investigations have been conducted so far to our knowledge into what extent chitinase activity is enhanced on NADES-pretreated substrate.

### 5.2. Chemical COS Synthesis

The chemical hydrolysis of chitin and chitosan has been examined thoroughly with various acids, among them hydrochloric acid, nitrous acid, phosphorous acid, sulphuric acid, lactic acid, formic acid, and trichloroacetic acid [35,133,134,135,136]. Additionally, reductive oxidants like hydrogen peroxide and potassium persulfate were applied to obtain water-soluble chitin derivatives [137,138].

Einbu and colleagues unraveled the acidic hydrolysis mechanism of HCl in detail, distinguishing between three general steps of depolymerisation, monomer production, and deacetylation, eventually yielding GlcN and acetic acid. Hydrochloric acid breaks GlcNAc–GlcN and GlcNAc–GlcNAc glycosidic bonds two to three orders of magnitudes faster compared to GlcN–GlcNAc and GlcN–GlcN links [34,133]. Possible explanations involve (a) the protective effect of the positively charged amino-group of GlcN against the protonation of the glycosidic oxygen and (b) the stabilisation of the oxocarbenium transition state by the N-acetyl group of GlcNAc. The initial enzyme hydrolysis rate is furthermore highly dependent on the substrate D_A_ and increases proportionally to a higher F_A_, while decreasing at longer reaction times due to deacetylation. The same study revealed how the degradation and accompanying N-deacetylation of chitosan took place in comparable rates in a diluted 3 M HCl solution, while the hydrolysis rate of glycosidic linkages was ten-fold faster than the rate of deacetylation in highly concentrated 12 M HCl. Trombotto et al. [139] were able to produce a homogenous series of chitosan-oligomers with a DP of 2-12 and a D_A_ between 0–90% by a two-step procedure: (i) the acid hydrolysis of fully de-N-acetylated chitosan (D_A_ 0) into COS of various DP and, after selective precipitations, (ii) the partial re-N-acetylation of those well-defined chito-oligomers dissolved in H_2_O/MeOH (50% each) with the addition of stoichiometric amounts of acetic anhydride (Ac_2_O) to obtain the expected D_A_.

Nonetheless, severe drawbacks for the chemical processing of COS remain, such as a wide DP range of resulting COS, including secondary compounds that are cumbersome to isolate, low yields, prevalent monomeric (low quality) products, residual acidity, and resulting toxicity of waste products, potential structural alterations of products, and high costs [35,36,140].

### 5.3. Biotechnological Chitin Pretreatment

To meet the challenge of the recalcitrant 3-D structure of chitin fibrils in a more sustainable way, enzymatic and fermentation pretreatments are the techniques of choice.

As alluded above, LPMOs may facilitate chitin hydrolysis through oxidation of crystalline chitin, therefore increasing the accessibility for chitinases. This synergistic effect represents untapped potential as a chitin pretreatment method for COS production, preferably in a one-pot approach. The focus of most studies was to discover new enzymes and to understand the mechanisms involved in LPMO substrate oxidation, rather than applying the AA family members in large-scale chitin conversion experiments. For lignocellulose containing substrates like wheat straw, corn stover, poplar, and wood, several research groups illustrated how the addition of LPMOs to commercial cellulolytic cocktails increased the saccharification yield by around 20–30% in average, given that oxygen and sufficient reducing agents to drive the reaction were present [109,141,142].

Wang and colleagues pretreated crab shells mechanically, on the one hand obtaining crude (600 µm) and ultramicro grinded (100 µm) particles, and on the other hand with an additional Alcalase 2.4L FG commercial protease mix [143]. Unfortunately, only the fine particles hereby received this treatment. Further enzymatic hydrolysis with the heterologously expressed *Bacillus subtilis* subsp. *Niger* chitinase *Bs*Chi were shown to be enhanced by 242%, in comparison to untreated substrate and mechanically treated crab shells (only 81% improvement).

Zhang et al. fermented chitin powder with *Chitinolyticbacter meiyuanensis* SYBC-H1 prior to hydrolysis and observed a transformation of the crystalline substrate towards a fleecy, fiber-like structure with diameters ranging from 10–200 nm [144]. Moreover, the reduction of the M_w_ and crystallinity was supported by X-ray diffraction and size exclusion chromatography analysis. When applying 8 U of purified *Chitinolyticbacter meiyuanensis* SYBC-H1 chitinase to a 50 mL reaction system with 20 g L^−1^ of the amorphous, self-proclaimed CBF chitin, 19.2 g L^−1^ of monomeric GlcNAc was obtained with a yield of 96% within 6 h.

### 5.4. Biotechnological COS Synthesis

Biologically active COS or paCOS can be produced enzymatically through (1) depolymerisation of pretreated chitin or regular chitosan with the corresponding glycosidic hydrolases chitinase and chitosanase. This yields mainly trimeric to monomeric glucosamines eventually, while statistical mixtures of higher DP product are obtained through the modulation of incubation time in the case of chitin [80]. Enzymatic degradation of partially acetylated chitosan polymers, however, tends to attain longer paCOS with mixed P_A_, D_A_, and DP and a higher likelihood of bioactivity [34,145]. (2) In vitro polymerisation of chito-oligomers using hydrolases with transglycosylating activity, resulting in high DP COS [146,147]. (3) De- or re-N-acetylation of chitooligosaccharides, deploying chitin- or chitooligosaccharide-deacetylases to introduce specific P_A_ and D_A_ of choice [94,145], or (4) in-vitro synthesis of defined chito-oligomers with a heterologous system, expressing chitinsynthases and CDA from fungi [148].

Chitinolytic enzymes have a high substrate subsite specificity and clearly defined catalytic activity, yielding partially defined products in a controlled reaction with less undesired by-products, contrary to acidic hydrolysis. Glycosyl hydrolases of the family 18, for instance, need an acetylated GlcNAc unit at the -1 subsite of the enzyme in order for them to break the glycosidic linkage of a GlcNAc but not GlcN unit, always resulting in a paCOS product with a GlcNAc at its reducing end [47,80]. A whole cluster of amino residues is involved in binding the long substrate polymers to the active enzyme site. The enzymatic cleavage of the glycosidic bond is defined to happen between the amino residues -1 and +1 of the active site, -n indicates the non-reducing end, whereas +n marks the reducing sugar end [149].

Pantaleone et al. showed furthermore, that chitosan is susceptible to a broad range of unspecific hydrolases, among them glycanases, proteases, lipases, and a tannase derived from various sources [150]. Other studies have successfully employed papain, pectinases, β-glucosidase, and cocktails containg α-amylase, proteinase, and protease [151,152,153,154]. Although hydrolytic activities towards chitosan might be caused by contaminations with chitosanases, and a rather large range of paCOS is obtained that way, crude enzyme mixes might represent economically feasible tools to produce chitosan-oligomers.

## 6. Quantitative and Qualitative Separation of COS Mixtures

The biological activity of COS is the sum of their structural properties, like M_w_, DP, D_A_, F_A_, and P_A_. Hence, the separation of hetero-chitooligomer mixtures according to their DP and single COS content are mandatory when trying to meet the high-quality standards necessary for biomedical applications and research. Additional qualitative analysis methods determining the secondary structural properties of COS, like D_D_ and P_A_, are key in understanding their effect mechanism.

Various procedures have been employed to recover and purify COS of common DP, amongst them gelfitration [155], ultrafiltration [156], and capillary electrophoresis (CE) with a laser-induced fluorescence detector [157]. The underlying principle of CE is that the amount of COS monomers present in acidic aqueous solution has an impact on the electrophoretic mobility. Although only small amounts of samples (5 µM) are required for high resolution analysis, the high material costs and the time-consuming process of derivatization with the fluorescence marker limit its large scale application severely. Nanofiltration has moreover been reported to distinguish between COS with a DP of 6–8 [158].

Chromatographic methods comprise immobilised metal anion chromatography for short COS with a DP of up to 4 (IMAC) [159], high-performance liquid chromatography (HPLC) with various detectors [157,160,161], and hydrophilic interaction chromatography (HILIC) coupled with an evaporative light-scattering detector (ELSD) [162]. Size-exclusion chromatography was successfully used to separate COS with a similar DP of -20 according to their length, independent from their D_D_ and P_A_, chaining SuperDex30 (GE Healthcare) columns and is typically the method of choice [163]. Aside from that, Ion-exchange chromatography (IEX) or cation-exchange chromatography can be applied to separate COS because the positively charged amino groups of deacetylated sugars interact with the negatively charged ion-exchange column packing material [164]. Further, hydrophilic interaction/weak cation-exchange mixed-mode chromatography (HILIC/WCX) [165] and mass spectroscopy coupled with a hybrid Q-TOF analyser [166] have been deployed. An immobilized lysozyme affinity chromatography, targeting the acetyl-group, proved useful in separating fully de-N-acetylated chitosan fragments with an average DP of 22 [167]. Continuous chromatography application or up-scaling is restricted by the necessity of column packing cleaning in between measurements, small analyte amounts at a time, and high production costs.

HPLC is probably the most frequently applied analysis method to gain structural insights of COS. Due to the acetyl group, a UV-light detector can be used to quantify GlcNAc units through monitoring of the absorbance rate at around 210 nm with an amino column [168], a reversed-phase column [169], or a carbohydrate column [170,171]. Chitosan-derived COS with a high D_D_ can only be analysed by this method if the analytes are derivatized with a chromophore in a strenuous process, since an absorbing element would be missing otherwise [172]. Alternatively to UV-light detectors, refractive index (RI) detectors typically coupled to amino columns [155] or size-exclusion columns [133] are resorted to, since they are able to identify both GlcN and GlcNAc subunits. Drawbacks of this methods are a low sensitivity and the inability to be employed for gradient-chromatography; furthermore, due to the high polarity of COS, the retention interaction with column material is rather weak and expensive equipment and solvents are generally required for HPLC. Further, the resolution decreases with the increase of COS chain length.

HILIC represents a method to separate compounds according to their polarity and is especially suitable for carbohydrates. Jiang et al developed an effective COS separation method with a stationary maltose-bond HILIC phase but recognized the impracticability of this method for large-scale applications due to the low solubility of high M_w_ COS in organic solvents [173]. Dong et al. developed a mixed HILIC/WCX approach, however, especially when analysing COS with high DP, the resolution of cation-exchange columns became increasingly inaccurate [165,173].

High performance anionic exchange chromatography with pulsed amperometric detection (HAPAEC PAD) was validated as a sensitive tool to distinguish de-N-acetylated glucosamines with DP 1–6 rapidly and with low sample concentrations [171]. Nevertheless, no reliable predictions can be made for acetylated glucosamines or COS above DP 6 with this technique.

Hamer et al. introduced novel P_A_ into (GlcNAc)_5_ with two deacetylases and were able to illustrate this by means of UHPLC coupled to an evaporative light-scattering detector (ELSD) and an electrospray ionization mass spectrometry (ESI–MS detector) [174].

To confirm the existence of an acetyl group or rather, a lack thereof, at the reducing and non-reducing sugar ends, as well as neighbouring variants, nuclear magnetic resonance (NMR) is employed. Studies have made use of both ^1^H NMR [92,133] and ^13^C NMR [7,175] to obtain sequence information of short COS of up to DP 5. Since the acquired information is merely limited to the average frequency of diads and triads, no definitive structure for COS mixtures can be obtained.

The matrix-assisted laser desorption/ionization time-of-flight mass spectrometry (MALDI-TOF MS) is another common method to identify COS according to their mass and charge of ions [139,176,177]. Besides the DP itself, information about the residue distribution within chito-oligomers of consistent DP can be derived as a function of D_A_ [139,177].

Recently Jiang et al. reported the development of two inexpensive, accurate, and easy to perform methods to determine the D_D_, namely an acid-base titration with bromocresol green in addition to a first-order derivative UV spectroscopy. The viability of their methods was supported furthermore by the comparison with ^1^H NMR [178].

An overview of both the preparative and analytical methods is provided in Table S1 and Table S2, respectively.

## 7. Perspectives and Conclusions

Despite substantial advancements in the field of biotechnological conversion of chitin in the last decades, chemical approaches reign supreme over enzymatic approaches on an industrial level. Green technologies are held back by excessive production costs of high-quality enzymes and the lower yields in comparison to chemical means of processing. The high crystallinity levels of native chitin in aqueous solution pose an addition challenge that requires a more sustainable pretreatment strategy in the long term. While some processive-acting chitinases, e.g., from the well-understood model organism *S. marcescens*, can depolymerise crystalline chitin, this ability comes with the cost of low conversion rates [50].

To overcome low yields, one promising route is to genetically engineer and thus optimize chitinolytic enzymes. Limón et al. illustrated how the fusion of a cellulose-binding domain to the fungal *Trichoderma harzianum* chitinases Chit33 and Chit42, respectively, increased the substrate-binding capacities which led to higher depolymerisation rates and anti-fungal activities [179,180]. Following studies deepened our understanding of the importance of carbohydrate-binding domains and enzyme–substrate interaction strength for the conversion of recalcitrant chitin [181,182,183].

Directed evolution has also been identified as an efficient method to generate chitinases with enhanced properties. Through error-prone PCR and DNA shuffling, mutant gene libraries are assembled and sequentially screened for chitinolytic activity. Fan et al. targeted the fungal *Beauveria bassiana* chitinase Bbchit1 using this method. Unexpectedly, they observed higher conversion rates for two Bbchit1 variants with point mutations outside the enzymatic substrate binding site [184]. Songsiriritthigul et al. proceeded similarly by mutant library construction and DNA shuffling of two chitinase genes from *B. licheniformis* with 99% identical sequence, obtaining one mutant with up to 2.7-fold improved activity towards colloidal chitin in comparison to wild-type variants, depending on pH [185].

A successful COS in vitro synthesis will most likely consist of several different enzymes combined, yet the complex enzymatic interactions of such a multi-step approach is still poorly understood and has to be further elucidated. Mekasha et al. examined the optimal enzyme ratios of a five mono-component cocktail for the complete saccharification of shrimp and crab chitins in their pioneering work. The cocktail incorporated the *S. marcescens* chitinases *Sm*ChitA, *Sm*ChitB, and *Sm*ChitC, a LPMO, and a β-N-acetylhexosaminidase [186]. The optimised cocktail yielded significantly higher chitin saccharification yields (70%) in comparison to a minimal *Sm*ChitA and *Sm*ChitB cocktail (40%). Moreover, different *Sm*ChitB to SmChitC ratios turned out to be beneficial for effective hydrolysation of the respective shrimp or crab chitins, while the *Sm*ChitA content of 40% was shared between both optimised cocktails. Further investigations into synergistic kinetics of chitin-targeting enzymes will be critical in developing a method to produce COS directly from crustacean shell wastes. One cannot emphasize enough the utmost importance of LPMOs for the degradation of crystalline chitin sources in the future, as they might represent the key protein class to solve both issues of recalcitrant substrate pretreatment and low efficiency of chitinases at the same time in a sustainable manner.

Intensive research efforts of the recent past allowed discovery of more chitinolytic enzymes, which also led to an increased understanding of their modes of action. As a result a new sort of novel class of chitinases has emerged—broad-specificity chitinases, which might contribute a lot to the cost reduction [187]. At present, only eight enzymes are reported to exhibit two to three different modes of chitin processing at the same time (with exochitinase, endochitinase, and β-N-acetylhexosaminidase activity being the different options). These can be categorised into single catalytic domain and multi-catalytic domain chitinases. Hence, either one catalytic site is able to perform multiple catalytic activities or several catalytic regions with distinct action modes toward chitin exist. In line with these findings, Hegsett et al. reported the *Streptomyces coelicolor* chitosanase A3(2) with potential activity towards all β-1,4-O-glycosidic linkages [87]. Further understanding of the mechanisms of broad-specificity glycosyl hydrolases like these might render the application of several abundant enzymes obsolete, therefore reducing production costs dramatically. To complicate things even more, chitinases of the GH18 family with secondary transglycosylating activities have been unravelled. Since COS production with the support of crude enzyme mixes or chitinases results in mostly small DP 1–3 fragments, TG-chitinases coupled with chitin deacetylases (CDA) might pose the missing link in recovering paCOS with higher M_w_ and a P_A_ and D_D_ of choice out of GlcNAc. Martinez et al. were able to supress the chitinolytic activity of a fungal *Trichoderma atroviride* chitinase with TG capabilities through site-directed mutagenesis [188]. This effort solved the persistent issue of product self-hydrolysation and represents an interesting new tool for COS engineering, as the in vitro polymerisation of longer chitin- and chitosan-oligomers circumvents the complex high DP COS production through hydrolases.

Another method, which was largely neglected with few exceptions [189,190], is the solid state fermentation. A recent study indicated that nearly dry conditions might be beneficial for the enzyme activity of chitinases, as this mirrors natural conditions for terrestrial chitinolytic organisms [189]. Therien and colleagues developed a self-proclaimed RAging method, in which they alternated between ball-mill grinding of shrimp and crab shells already containing a commercial chitinase mix, with resting periods at 45 °C for 20 times. Only those with diluted acetic-acid-pretreated chitin sources could be converted into GlcNAc with a conversion rate of at least 40% without the necessity of solvents.

Lastly, the center of attention has revolved around aerobic glycosyl hydrolase producers for a long time, while anaerobic organisms represent mainly unknown territory to date. This is despite the fact that aeration in industrial large-scale fermentations has to be carefully monitored and represents a cost factor, while very promising studies with anaerobic fermentations have been conducted [20] and the role of maritime organisms for the natural recycling of crustacean wastes is undisputed [191].

With the recent findings of more and more chitin-directed enzyme classes like TG-chitinases, broad specificity chitinases and chitosanases, LMPO, CDA, and COD, paired with an increased understanding of their mechanisms in addition to the development of cheap and easy analysis methods for COS [178], all parts of the puzzle seem to be complete. The last stepping stone for the sustainable conversion of natural chitin waste streams is to piece all these puzzles pieces together into a method that is suitable for large-scale applications. Regarding product specificity and quality, enzymatic approaches surpassed chemical means of chitin processing years ago, and the economic reasons that prevent the prevalent application of biotechnological methods in the industry might be resolved in the near future.

## Supplementary Materials

The following are available online at https://www.mdpi.com/1660-3397/18/2/93/s1.
