# Supplementary material for: Enzymatic Modification of Native Chitin and Conversion to Specialty Chemical Products
Source: Mar Drugs. 2020 Jan 30;18(2):93. doi: 10.3390/md18020093 (PMC7073968; doi:10.3390/md18020093)
Supplement: Supplementary file 1 [file marinedrugs-18-00093-s001.pdf]

Supplementary data

Table 1: Preparative methods to separate COS according to physiochemical properties.

**Methods to recover and isolate chitin derivatives**	**COS length (DP), or weight**	**Product quantity**	**Specifics**	**Reference**
Gel permeation chromatograpy (GPC) coupled with HPLC for analysis	DP 2 – 6	< 2 g	Separation according to M_W-dependent retention time using a pullulan standard.	(Choi, Ahn, Lee, Byun, & Park, 2002)
Ultrafiltration	5 – 30 kDa		High weight chitosan was fractionated.	(S. A. Lopatin, Derbeneva, Kulikov, Varlamov, & Shpigun, 2009)
Capillary electrophoresis	DP 2 - 6	< 5 µM	Very low detection limit, requires derivatisation with fluorescence marker.	(Hattori, Anraku, & Kato, 2010)
Nanofiltration	DP 6 - 8	16 L of < 5 % chitosan	Large scale preparative method, able to yield up to 82,2 % pure COS.	(H. Dong et al., 2014)
Immobilised metal anion chromatography (IMAC)	DP 2 - 4	< 40 mg	60 – 95 % yield with 95 % (DP 2 -3) and 90% (DP 4) purity.	(Le Dévédec et al., 2008)
High performance liquid chromatography (HPLC)<br>With amino-column + UV/RI detector<br>With reversed-phase column + UV detector<br>With carbohydrate-column + UV detector	 DP 2 - 6<br>DP 2 – 7<br>DP 1 - 8	 NA<br>300 mg/ml<br>< 2,2 mg/ml	UV detector: GlcN only detectable through coupling of a chromophore. RI-detector: GlcN and GlcNAc, low sensitivity. Generally: expensive, limited to low DP COS.	 (Lv et al., 2016)<br>(Sergey A. Lopatin et al., 1995)<br>(Jung, Souleimanov, Park, & Smith, 2007)
Hydrophilic interaction chromatography (HILIC) coupled with an evaporative light-scattering detector (ELSD)<br>HILIC with weak cation exchange chromatography mixed mode (WCX)	DP 2 - 6<br><br>DP 2 - 6	5 ml of 100 mg/ml<br>5 µl	High M_W chitosan is insoluble in organic solvents, no large scale application.<br>Resolution of ion exchange columns drops with increasing DP of COS.	(M. Jiang et al., 2014)<br><br>(X. Dong, Shen, Gou, Chen, & Liang, 2012)
Size exclusion chromatography (SEC)	DP 4 – 20	200 mg	COS separation independent of P_A and D_D.	(Sørbotten, Horn, Eijsink, & Vårum, 2005)
Ion-exchange chromatography (IEX)<br>High performance an-ionic exchange chromatography with pulsed amperometric detection (HAPAEC PAD)	DP 5<br>DP 1 - 6<br>DP 1 - 5	20 µg<br>< 10 mg/L<br>3 ml of 0,5% chitosan	Separation of D_2A_3 isobars according to P_A.<br>GlcN with DP < 6, no acetylated glucosamine detection.	(Haebel, Bahrke, & Peter, 2007)<br>(Cao et al., 2016)<br>(Santos-Moriano, Woodley, & Plou, 2016)
Immobilised lysozyme affinity chromatography	Mixture with DP 22 in average	20 mg	Acetyl-group dependent interaction, separates GlcN from GlcNAc.	(Sasaki, Kristiansen, Fukamizo, & Vårum, 2003)

Table 2: Analytical methods to analyse physiochemical properties or sequences of COS.

Methods to analyse and identify COS	COS length (DP), or weight	Product quantity	Specifics	Reference
UHPLC coupled to an evaporative light scattering detector (ELSD) and an electrospray ionization mass spectrometry (ESI–MS detector)	DP 1 - 6	1 µl of 1 mM solution	Information about the pattern of acetylation (P_A) within the COS.	(Hamer et al., 2015)
Mass spectroscopy coupled with a hybrid QTOF analyser	DP 1 - 4	NA	The DP, but not the P_A is retrieved.	(Santos-Moriano et al., 2016)
The matrix-assisted laser desorption/ionization time-of-flight mass spectrometry (MALDI-TOF MS)	DP 3 - 6<br><br>DP 3 - 7	1 µl<br><br>1 µL of 0,5 g/L COS	Information about DP and residue distribution within COS of consistent DP as function of D_A.	(Doan, Tran, Nguyen, Nguyen, & Wang, 2018)<br>(Trombotto, Ladavière, Delolme, & Domard, 2008)
^{1}H NMR<br><br>^{13}C NMR	DP 2<br><br>DP 30 in average<br>< 28,7 kg/mol<br><br>NA	15 mg/ml In DCl<br>NA<br><br>5 and 50 mg<br><br>NA	Information about F_A (^{1}H NMR) and P_A (^{13}C NMR); the frequency and nature of diads and triads is obtained. No definitive structure, but models of statistical distribution of diads can be calculated.	(Einbu & Vårum, 2007)<br><br>(Martinou, Bouriotis, Stokke, & Vårum, 1998)<br>(Weinhold, Sauvageau, Kumirska, & Thöming, 2009)<br>(Paul et al., 2015)
Acid-base tritration with bromocresol green and first order derivative UV spectroscopy	DP 2 – 20 with ≤1 kDa and ≤3 kDa samples	0,5 g and 0.1 g respectively	Information about degree of deacetylation (D_D) of COS.	(Y. Jiang et al., 2017)
